# Combating sepsis: bottlenecks to breakthroughs

**DOI:** 10.1016/j.lanwpc.2023.100923

**Published:** 2023-09-29

**Authors:** The Lancet Regional Health – Western Pacific

World Sepsis Day, occurring annually on September 13, is essential to raise global awareness of sepsis and remind us to review the way we combat it. Sepsis remains a major global health threat yet there is insufficient awareness. The enforcement of WHA70.7 Sepsis Resolution, a WHO resolution recognising sepsis as a global health priority in 2017, is surprisingly low. As of August, 2023, only 16 countries worldwide have prioritised sepsis in national health policies, strategies, or sepsis-related initiatives. Most of them are high-income countries (HICs) except for Thailand, the only low-income and middle-income country (LMIC) to have prioritised sepsis policies. Decades of research advances have greatly improved our understanding of the pathophysiology of sepsis and the progress in clinical practices has decreased its overall mortality. Combating sepsis relies on prevention, early diagnosis, and timely effective critical care management, which are hitting bottlenecks.

Public health measures to improve sanitation and water quality and availability are the most cost-effective way to prevent community-acquired sepsis. Nevertheless, it remains unaddressed in low-resource settings with heavy burden but poor hygiene practices. Vaccination is another promising strategy, yet the only vaccine currently available is the maternal immunisation with Group B streptococcus (GBS) vaccine during pregnancy to prevent neonatal sepsis. None of the GBS vaccine products had entered phase 3 trials as of April, 2022, and the strategy might not be feasible in many LMICs before antibiotic provision and systemic GBS screening programmes are properly addressed.

For diagnosis, there is an unmet need for establishing and validating sepsis screening systems in LMIC settings, as well as a lack of early and rapid diagnostic tools. The latest definition for sepsis, Sepsis-3, uses an increase in the Sequential (sepsis-related) Organ Failure Assessment (SOFA) score of 2 points or more as a clinical indicator. However, it was mainly developed in HICs and thereby its applicability in LMICs remain uncertain. A score has been developed based on the cohorts from sub-Saharan African countries—the Universal Vital Assessment score. Disappointingly, a recent comparative study found that it did not outperform other scores in LMICs. Biomarkers such as C-reactive protein, lactate, and procalcitonin could aid earlier recognition, but diagnostic biomarkers specific to sepsis have not yet been identified. Hopefully, innovative approaches are being developed to accelerate early diagnosis of sepsis. For example, machine learning techniques are utilised to develop individual-level surveillance algorithms for sepsis, create sepsis diagnostic tools, and identify biomarkers.

Novel therapeutics to treat sepsis are being developed but most are at the preclinical phase. The main strategy is to target the pathogenesis of sepsis to address the three principal defects during the septic process—epithelial barrier dysfunction, endothelial leakage, and disrupted cellular metabolism. Other strategies are immunotherapies to modulate immune imbalance. Further experimental research and better designed clinical trials are needed for breakthrough progress. Current treatments are also facing challenges. For bacterial sepsis, early antimicrobial treatment is essential to maximise survival but should be balanced against the increase in antimicrobial resistance. LMICs have a higher risk of antimicrobial resistance owing to limited access to the most appropriate antibiotics and early intervention in the absence of a clear diagnosis. Meanwhile, worrisome wide variations in standard of care for sepsis diverging from WHO guidelines were observed in a global neonatal sepsis observational study—more than 200 different antibiotic combinations were used, with frequent switching of antibiotics, leading to a substantial burden of antimicrobial resistance and high mortality in patients with neonatal sepsis. Thus, beyond optimising antimicrobial timing and spectrum, improving guideline adoption and compliance to care protocols is also crucial to address antimicrobial resistance during sepsis management. Sepsis caused by fungi or viruses is associated with poor outcomes but is often neglected. In particular, management of virus-induced sepsis is absent from guidelines and should be considered in patients with sepsis who are underdiagnosed. Another challenge of sepsis management is post-sepsis syndrome. For the first time, assessment and follow-up for long-term physical impairments, neurocognitive impairments, and mental health disorders after hospital discharge was recommended in the latest International Guidelines for Management of Sepsis and Septic Shock 2021. However, there are scarce data worldwide regarding the burden and mechanisms of post-sepsis syndrome, let alone specific guidelines for optimal care in health care or the community setting.

To achieve breakthroughs in fighting sepsis, we need to establish consistent and updated criteria through incorporating high-quality data from LMICs, implement effective and feasible early diagnostic tools, and most importantly, achieve fundamental advances in therapeutic strategies by addressing antimicrobial resistance, post-sepsis syndrome, and exploring targeted treatments.

